# Light and Dehydration but Not Temperature Drive Photosynthetic Adaptations of Basal Streptophytes (*Hormidiella*, *Streptosarcina* and *Streptofilum*) Living in Terrestrial Habitats

**DOI:** 10.1007/s00248-018-1225-x

**Published:** 2018-07-04

**Authors:** Mattia Pierangelini, Karin Glaser, Tatiana Mikhailyuk, Ulf Karsten, Andreas Holzinger

**Affiliations:** 1 Department of Botany, Functional Plant Biology, University of Innsbruck, 6020 Innsbruck, Austria; 2 Present Address: Laboratoire de Génétique et Physiologie des microalgues, InBioS/Phytosystems, Institut de Botanique, Université de Liège, Liege, 4000 Belgium; 3 Applied Ecology and Phycology, Institute of Biological Sciences, University of Rostock, Albert-Einstein-Strasse 3, 18059 Rostock, Germany; 4 M.G. Kholodny Institute of Botany, National Academy of Sciences of Ukraine, Tereschenkivska Str. 2, Kyiv, 01004 Ukraine

**Keywords:** Acclimation, Desiccation, Green algae, Photoprotection, Streptophyta, Temperature

## Abstract

Streptophyte algae are the ancestors of land plants, and several classes contain taxa that are adapted to an aero-terrestrial lifestyle. In this study, four basal terrestrial streptophytes from the class Klebsormidiophyceae, including *Hormidiella parvula*; two species of the newly described genus *Streptosarcina* (*S. costaricana* and *S. arenaria*); and the newly described *Streptofilum capillatum* were investigated for their responses to radiation, desiccation and temperature stress conditions. All the strains showed low-light adaptation (I_k_ < 70 μmol photons m^−2^ s^−1^) but differed in photoprotective capacities (such as non-photochemical quenching). Acclimation to enhanced photon fluence rates (160 μmol photons m^−2^ s^−1^) increased photosynthetic performance in *H. parvula* and *S. costaricana* but not in *S. arenaria*, showing that low-light adaptation is a constitutive trait for *S. arenaria.* This lower-light adaptation of *S. arenaria* was coupled with a higher desiccation tolerance, providing further evidence that dehydration is a selective force shaping species occurrence in low light. For protection against ultraviolet radiation, all species synthesised and accumulated different amounts of mycosporine-like amino acids (MAAs). Biochemically, MAAs synthesised by *Hormidiella* and *Streptosarcina* were similar to MAAs from closely related *Klebsormidium* spp. but differed in retention time and spectral characteristics in *S. capillatum*. Unlike the different radiation and dehydration tolerances, *Hormidiella*, *Streptosarcina* and *Streptofilum* displayed preferences for similar thermal conditions. These species showed a temperature dependence of photosynthesis similar to respiration, contrasting with *Klebsormidium* spp. and highlighting an interspecific diversity in thermal requirements, which could regulate species distributions under temperature changes.

**Electronic supplementary material:**

The online version of this article (10.1007/s00248-018-1225-x) contains supplementary material, which is available to authorized users.

## Introduction

Streptophyte green algae separated from the Chlorophyta lineage more than 700 million years ago (MYA) [1, 2]. Following this split, streptophytes diversified into six paraphyletic classes, the basal (Mesostigmatophyceae, Chlorokybophyceae, Klebsormidiophyceae) and advanced streptophytes (Charophyceae, Coleochaetophyceae, Zygnematophyceae), which originated the land plants about 450–500 MYA [1, 3, 4]. Although, the position of Mesostigmatophyceae and Chlorokybophyceae within Streptophyta has recently been debated [5, 6], members of the Klebsormidiophyceae are considered as a basal lineage of Streptophyta [6], whilst the Zygnematophyceae are currently believed to be the sister group of land plants [7, 8]. In the present study, three members of basal terrestrial streptophytes, including the newly described genera *Streptosarcina* Mikhailyuk et Lukešová (sister lineage to *Hormidiella* Iyengar et Kanthamma) and *Streptofilum* Mikhailyuk et Lukešová (separate lineage inside Streptophyta), as well as a new epitype strain of *Hormidiella parvula* Iyengar et Kanthamma [9] were investigated.

In addition to their key phylogenetic position, basal streptophytes show adaptations to the hydrological gradient separating freshwater algae from land plants [10–12]. Several basal streptophytes are typically found in cryptogamic covers on rocks and tree bark, and in biological soil crusts worldwide [13–16]. Due to their cosmopolitan distribution, these algae are considered pioneers of empty ground, and hence good models to study their interaction with terrestrial habitats, for example during soil colonisation, as well as how this interaction may have developed during the early stages of land colonisation by plants in the Middle Ordovician Period [17].

Due to the absence of light attenuation by water [18], it has been suggested that algae colonising terrestrial environments became exposed to high photosynthetically active radiation (PAR) [11, 12, 19, 20]. However, until now, the photosynthetic apparatus of several terrestrial basal streptophytes (e.g. *Klebsormidium* Silva, Mattox et Blackwell) has been described as having low-light adaptation traits, with initial values for light-saturating photosynthesis (I_k_) often below 80 μmol photons m^−2^ s^−1^ [16, 21, 22]. Unexpectedly for such low-light-adapted species, a lack of photoinhibition at photon fluence rates as high as 1500 μmol photons m^−2^ s^−1^ has been observed in *Klebsormidium* spp. [22–24]. This tolerance can be related to the presence of photoprotective mechanisms, for example non-photochemical quenching (NPQ), which allows excess light absorbed to be dissipated as heat, preventing the formation of reactive oxygen species (ROS) and photooxidative damage [25, 26]. NPQ is present in all photosynthetic organisms, although with differences in the mechanisms involved [27, 28]. For instance, in green algae, including some streptophytes, the energy-dependent component qE is the major contributor to NPQ [23]; for others, such as some members of Bryopsidales (Ulvophyceae), it is not [29, 30]. The qE component is triggered by the formation of a trans-thylakoid proton gradient and requires a protein sensor of the lumen pH, which in vascular plants and advanced streptophytes is the Photosystem II Subunit PSBS [23, 31], and in chlorophytes, such as *Chlamydomonas* and basal streptophytes, is the light-harvesting stress-related complex LHCSR (possibly in combination with PSBS) [23, 32, 33].

In addition to PAR, ultraviolet radiation (UVR) is also part of the natural solar spectrum. Particularly, UV-B (280–315 nm) plays a critical role for phototrophs, because these high-energy wavelengths may lead to molecular and biochemical disturbances in cells, such as DNA damage or induction of ROS [34, 35]. The physiological consequences are manifold and include, for example strong reductions in photosynthesis [36, 37]. To counteract UVR damage, many terrestrial algae biosynthesise and accumulate mycosporine-like amino acids (MAAs) as sunscreen compounds [38–40], which absorb these short wavelengths followed by re-emission as harmless heat, thereby shielding intracellular structures and biomolecules [41]. The presence of a range of mechanisms to cope with concomitant high PAR and UVR thus raises the question if the low-light requirements for photosynthesis observed in many terrestrial streptophytes are constitutive traits or, rather, reflect physiological plasticity and a (low) light-acclimated status of the cells.

Besides irradiance, dehydration plays a pivotal role in shaping photosynthesis of terrestrial microalgae. In the case of cyanobacteria, dehydration tolerance has been related to the presence of different protective mechanisms in the photosynthetic apparatus, which reduce ROS production [42, 43]. In desiccation-tolerant green algae, the photosynthetic apparatus is rapidly inactivated when intracellular water levels drop below a critical threshold [44, 45]. Yet, during the dehydration phase, exposure to relatively high photon fluence rates compromise the photosynthesis and the ability of the cells to recover when water is restored [22, 46]. For *Klebsormidium*, dehydration was suggested to act as a selective force shaping the adaptation of this organism to low light [22].

As well as the necessity of dealing with changes in irradiance and water availability, algae dwelling in terrestrial habitats must also cope with fluctuations of temperature [47]. Previous investigations have shown that the photosynthetic and respiratory responses of some Klebsormidiophyceae to short-term rising temperatures [13, 21, 48, 49] are similar to those of other microalgae, where the two metabolic processes have different temperature requirements [50–52]. However, recently, for communities in geothermal streams and a species of marine diatom, it has been observed that the short-term sensitivity to temperature is not always different for photosynthesis and respiration [53, 54]. This emerging interspecific diversity in thermal sensitivity has been overlooked for terrestrial microalgae (as well as for many species that dominate in phytoplankton communities). However, it might be crucial to understand species adaptations to different thermal environments, and/or community composition shifts under temperature changes.

In this study, we aimed to understand the photosynthetic adaptations employed by basal streptophytes to successfully colonise terrestrial habitats. We have taken advantage of newly described genera and species of basal streptophytes [9], which should broaden our understanding of the physiological behaviour of ancestral streptophytes. We have emphasised species photosynthetic traits, light acclimation abilities and protective mechanisms (against PAR and UVR). We hypothesised that light-adaptation traits are coupled to dehydration tolerance. Finally, we investigated the temperature requirements for photosynthesis and respiration, to test if the two processes have the same or different temperature dependence.

## Materials and Methods

### Organisms and Culture Conditions

Five unialgal streptophytes isolated from terrestrial habitats in different biogeographical regions were investigated: *Hormidiella parvula* (Wyoming, USA), *Streptofilum capillatum* Mikhailyuk et Lukešová (Czech Republic), *Streptosarcina arenaria* Mikhailyuk et Lukešová (strain Prim-3-3, Ukraine, and strain AL-63, Slovakia) and *Streptosarcina costaricana* Mikhailyuk et Lukešová (Costa Rica) [9]. All strains are deposited in the public Algal Culture Collection at the University of Göttingen, Germany (SAG) as *H. parvula* (SAG 2558), *S. capillatum* (SAG 2559), *S. arenaria* (strain Prim-3-3 is SAG 2562 and strain AL-63 is SAG 2560), and *S. costaricana* (SAG 36.98) [9]. For experiments, species were grown in modified Bold’s basal culture medium (3 N BBM) [55], buffered at pH 7.5 using 40 mmol L^−1^ HEPES. Cultures were incubated in a growth chamber with a light:dark cycle of 16:8 h under control light conditions (CL; 60 μmol photons m^−2^ s^−1^, provided by FL40SSW/37 light tubes) and with a temperature of 15 °C during the dark phase and 20 °C during the light phase. Photoacclimation was studied by exposing algal cells to a moderate, higher photon fluence rate (ML; 160 μmol photons m^−2^ s^−1^) for 1 week, during the light cycle. All species were maintained in batch growth, using 200-mL Erlenmeyer flasks filled with a maximal culture volume of 100 mL. To avoid nutrient depletion and to maintain low cell concentrations, cultures were refreshed with culture medium every week, which guaranteed continuous log-phase growth.

### Rapid Light Curves and NPQ Measurements

The photosynthetic characteristics and NPQ capacities of the four species were analysed using a PAM 2500 fluorimeter (Heinz Walz, Effeltrich, Germany) according to Pierangelini et al. [22], with a few modifications. Rapid light curves (RLCs) were obtained by exposing cells to photon fluence rates between 0 and 2014 μmol photons m^−2^ s^−1^. To allow an active Calvin-Benson cycle and to avoid the potential occurrence of photoinhibition related to a slow NPQ activation, during the RLC, the photon fluence rate was increased every 90 s [22, 56]. Fluorescence induction curves for NPQ estimation were obtained by exposing cells to 20 saturating light pulses (300 ms) of an actinic light of 618 μmol photons m^−2^ s^−1^, followed by a dark recovery time to monitor the NPQ relaxation phase. Before both the RLC and NPQ measurements, cells were kept in the dark for 15 min.

### SDS-PAGE and Western Blotting

The presence of the LHCSR3 protein in *S. arenaria* (strain SAG 2562) and *S. costaricana* was assessed through the western blot technique, following the procedure described by Roach and Na [57]. These two species were selected for LHCSR3 detection because they showed the highest and lowest NPQ, respectively, under controlled light conditions (Table 1). For both isolates, protein extracts from cultures with different amounts of Chl *a* (~0.62, 0.31 and 0.16 μg) were loaded in different lanes of the gel. The presence of a sufficient amount of protein in the lanes was assessed through staining with Coomassie Brilliant Blue. Proteins extracted from *Chlamydomonas reinhardtii* Dangeard were used as control. The blots were then probed with an antibody against LHCSR3 (catalog no. AS14 2766; Agrisera, Sweden), which was kindly provided by Thomas Roach (University of Innsbruck, Austria).

**Table 1:** Photosynthetic characteristics (RLC curves) and maximal NPQ capacities of the five basal streptophyte green algae grown under control (60 μmol photons m^−2^ s^−1^) and moderate (160 μmol photons m^−2^ s^−1^) light intensities

Species	α^a^	rETR_max_^b^	I_k_^c^	β^a^	NPQ_max_	*F*_*v*_/*F*_*m*_
60 μmol photons m^−2^ s^−1^						
*H. parvula*	0.328 (0.018)	19 (4)	57 (12)	− 0.002 (0.004)	1.94 (0.19)	0.68 (0.01)
*S. arenaria* SAG 2562	0.395 (0.026)	13 (2)	34 (2)	− 0.001 (0.002)	2.61 (0.32)	0.66 (0.00)
*S. arenaria* SAG 2560	0.353 (0.043)	24 (4)	67 (5)	− 0.007 (0.004)	1.58 (0.22)	0.66 (0.06)
*S. costaricana*	0.361 (0.017)	17 (4)	46 (8)	− 0.008 (0.003)	0.93 (0.12)	0.66 (0.04)
*S. capillatum*	0.379 (0.052)	18 (3)	47 (2)	− 0.006 (0.000)	1.32 (0.21)	0.72 (0.03)
160 μmol photons m^−2^ s^−1^						
*H. parvula*	0.221 (0.031)*	27 (3)*	124 (31)*	− 0.001 (0.005)	2.02 (0.49)	0.57 (0.04)*
*S. a.* SAG 2562	0.284 (0.024)*	14 (2)	51 (12)	0.001 (0.003)	2.15 (0.34)	0.60 (0.04)*
*S. a.* SAG 2560	0.326 (0.019)	21 (1)	66 (2)	− 0.005 (0.001)	1.40 (0.31)	0.67 (0.00)
*S. costaricana*	0.268 (0.045)*	26 (2)*	98 (26)*	− 0.002 (0.003)	1.32 (0.21)*	0.59 (0.05)

Asterisks indicate statistically significant differences of the parameters measured at moderate light in comparison to the control. Values in brackets represent standard deviation (*n* ≥ 3)

^a^electrons photons^−1^

^b^μmol electrons m^−2^ s^−1^

^c^μmol photons m^−2^ s^−1^

### Effects of UVR on Sunscreen Induction and Accumulation

The streptophyte algal strains cultured for 3 days under the above conditions were transferred to 600-mL glass Petri dishes, provided with new medium and kept at around 23 °C for 4 days under one of two radiation conditions, with a 16:8-h light:dark cycle of PAR only (400–700 nm) or PAR + UVR (PAR + UV-A + UV-B, 295–700 nm). In both the control and UV treatments, Lumilux Deluxe Daylight L15W/950 (OSRAM) provided 80–90 μmol (PAR) photons m^−2^ s^−1^. The additional UVR was emitted by Q-Panel-UVA 340 fluorescent lamps (Q-Panel, Cleveland, OH, USA). Whilst the control was covered with a 400-nm cutoff filter foil (Folex PR; Folex, Dreieich, Germany), resulting in total UV-A/B elimination, the UV-treated algal cultures were exposed to 6–7 W m^−2^ UV-A and 0.37–0.45 W m^−2^ UV-B under a 295-nm cutoff filter (Ultraphan UBT 295; Digefra, Fürstenfeldbruck, Germany). PAR/UVR was measured with a Li-Cor LI-190-SB cosine corrected sensor connected to a Li-Cor LI-1000 data logger (Lambda Instruments, Lincoln, NE, USA) and with a PMA broadband radiometer (Solar Light Co., Philadelphia, PA, USA). After the treatment, algal biomass was harvested by filtration (pre-weighed GF 6 filters; Carl Roth, Karlsruhe, Germany), dried at 30 °C overnight and weighed again to determine dry weight. This biomass was extracted and further processed for HPLC analysis as described in detail by Karsten et al. [58]. Samples were analysed with an Agilent HPLC system (Agilent, Waldbronn, Germany), and MAAs were separated on a stainless-steel Phenomenex Synergi Fusion RP-18 column (4 μm, 250 × 3.0 mm I.D.) protected with a RP-18 guard cartridge (20 × 4 mm I.D.) of the same material (Phenomenex, Aschaffenburg, Germany). The mobile phase was 2.5% aqueous methanol (*v*/*v*) plus 0.1% acetic acid (*v*/*v*) in water, run isocratically at a flow rate of 0.5 mL min^−1^. MAAs were detected online with a photodiode array detector at 330 nm, and absorption spectra (290–400 nm) were recorded every second, directly on the HPLC-separated peaks. Identification and quantification were done by spectra, retention time and co-chromatography with extracts of closely related *Klebsormidium* species, according to Kitzing et al. [38]. Due to the small amount of biomass, only one replicate for each strain could be tested, and hence, the calculated concentrations indicate only a trend. Nevertheless, the qualitative aspect of the data is reliable.

### Dehydration and Rehydration Experiments Monitored by the Effective Quantum Yield of PSII

The effect of dehydration and rehydration on the effective quantum yield of PSII (YII) was determined using the standardised approach with specially designed desiccation chambers [48]. Algal biomass (~1–2 mg Chl *a* L^−1^) resuspended in 200-μL 3 N BBM was transferred to Whatman GF/F glass-fibre filters (Whatman, Dassel, Germany) (*n* = 4) and placed in desiccation chambers filled with 100 g silica gel, which resulted in a relative air humidity (RH) of ~10% inside the chamber. The RH was recorded with a PCEMSR145STH mini data logger (PCE Instruments, Meschede, Germany), and the chambers were exposed to constant photon fluence rates (40 μmol photons m^−2^ s^−1^) at ambient room temperature (23 ± 0.5 °C). This light level was chosen to avoid potential damage from high light exposure [22, 46] and to assess the light-independent ability of species to tolerate dehydration events. During the dehydration and recovery processes, the YII was regularly measured through the transparent top lid of the chambers, using a PAM 2500 fluorimeter (Heinz Walz GmbH, Effeltrich, Germany) [48]. The distance between the PAM light probe and the algae on the filters was kept constant (12 mm). Once the YII of each replicate of the individual algal species reached 0, the dehydration period was ended. This was immediately followed by rehydrating the algae on the filters with 200 μL of 3 N BBM and placing them in a chamber containing 100 mL tap water (RH ~96%) to follow the YII recovery.

### Response of Photosynthesis and Dark Respiration to Rapid Increase of Temperature

**Fig. 1:**
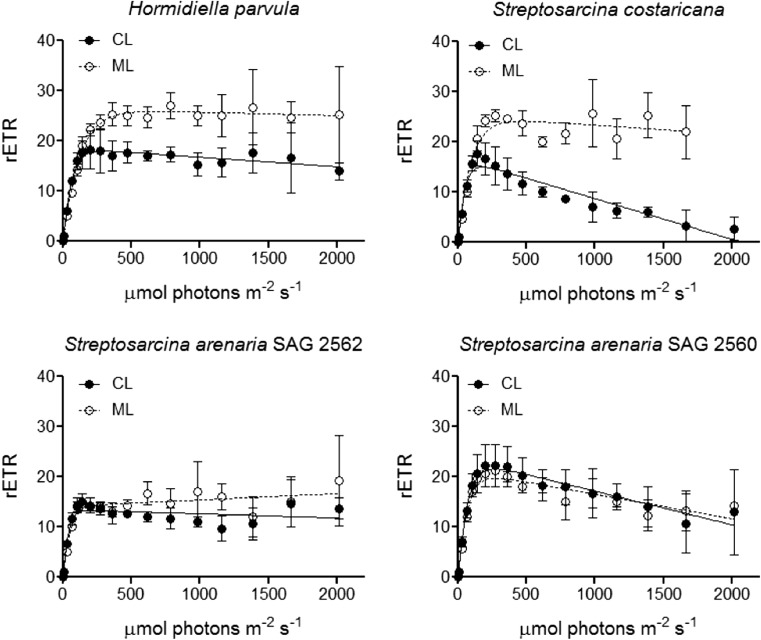
Photosynthetic response to irradiance (RLC curves) of *Hormidiella parvula*, *Streptosarcina costaricana* and *Streptosarcina arenaria* strains SAG 2562 and SAG 2560 cultured in control (CL; 60 μmol photons (PAR) m^−2^ s^−1^) and moderate [ML; 160 μmol photons (PAR) m^−2^ s^−1^] photon fluence rates. Vertical bars indicate standard deviations of at least three independent culture replicates

The short-term responses of photosynthetic oxygen evolution and respiratory oxygen consumption (dark respiration) to increasing temperatures were assessed following the procedure of Karsten and Holzinger [13]. For the experiments, cells were harvested from the culture (in CL light), resuspended in 3 mL of fresh 3 N BBM medium containing 3 mmol L^−1^ of NaHCO_3_, and placed in a 3-mL thermostatic acrylic chamber (type DW1; Hansatech Instruments, Norfolk, UK) to which a Presens Fibox 3 oxygen optode (Presens, Regensburg, Germany) was fitted. Samples were then exposed to rising temperatures from 5 to 45 °C. At each temperature, cells were initially incubated in the dark for 30 min, and the last 10 min of this incubation period was used to measure the respiration. After the dark period, cells were exposed to 185 μmol photons m^−2^ s^−1^ for 10 min, with the final 5 min used to calculate the photosynthetic oxygen evolution. This photon fluence rate was chosen because it was expected to saturate electron transport rates (see RLCs; Fig. 1). To prevent photorespiration, during the experiments, the O_2_ concentration in the chamber was maintained below air saturation. The rates of respiration and photosynthesis were both normalised to Chl *a*, extracted as in Pierangelini et al. [22]. Gross photosynthesis was calculated as the sum of respiration and net photosynthesis. To calculate the activation energy (*E*_*a*_), the deactivation energy (*E*_*h*_) [the temperature at which half of the enzymes are inactive (*T*_*h*_)], for both photosynthesis and respiration, ln(gross P) and ln(R) values were fitted through the modified Sharpe-Schoolfield equation for high-temperature inactivation, using the nonlinear least squares regression with the ‘nlsLM’ function in the ‘minpack.lm’ package in R software (R Core Team 2017; v3.2.2), as described by Padfield et al. [51, 53]. Optimal temperatures (*T*_opt_) for photosynthesis and respiration were calculated with Eq. 2 in Padfield et al. [51].

### Statistical Analyses

The results are expressed as the mean value of at least three independent culture replicates ± standard deviations. We tested the significance of the mean differences amongst the four species using one-way ANOVA, followed by Tukey’s multiple comparison test. The variation amongst means in relation to temperature and species was tested by using two-way ANOVA followed by Bonferroni post-test. Comparisons between two single data points were performed by two-tailed *t* test. All the analyses were performed using the software GraphPad Prism 5, setting the threshold of significance at 95%.

## Results

### Photosynthetic Characteristics Under Different Light Regimes

The results of the RLCs, reflecting the response of the photosynthetic apparatus to a short-term increase of light, are shown in Table 1 and Fig. 1. In control light (CL), the ability to harvest light (α) did not differ amongst the four species (one-way ANOVA, *p* = 0.2387). All the species showed similar maximal electron-transport capacity (rETR_max_), although for *S. arenaria* SAG 2562, the ETR_max_ was slightly lower in comparison to *S. arenaria* SAG 2560 (one-way ANOVA, *p* = 0.0445). Although statistically weak (one-way ANOVA, *p* = 0.0653), *S. capillatum*, *S. arenaria* SAG 2560 and *S. costaricana* showed higher photoinhibition (β) levels than *H. parvula* and *S. arenaria* SAG 2562. Acclimation to moderate light (ML) induced species-specific changes in the photosynthetic-apparatus performance (Fig. 1 and Table 1). For *H. parvula* and *S. costaricana*, acclimation to ML was related to a lower α (*t* test, *p* = 0.0068; *p* = 0.0277), higher rETR_max_ (*t* test, *p* = 0.0408; *p* = 0.0226) and I_k_ values twice as high as in the CL (*t* test, *p* = 0.0247; *p* = 0.0311). In comparison, *S. arenaria* showed less photoacclimation capability*.* For *S. arenaria* SAG 2562, exposure to ML lowered α (*t* test, *p* = 0.0060) but did not modulate rETR_max_ (*t* test, *p* = 0.6623) and I_k_ (*t* test, *p* = 0.0810). For *S. arenaria* SAG 2560, no acclimation was observed, and this strain maintained the same photosynthetic capacity under both growth-light regimes. For *S. capillatum*, the performance under ML could not be determined, as the cultures died for reasons unrelated to the light treatment.

### Non-Photochemical Quenching

**Fig. 2:**
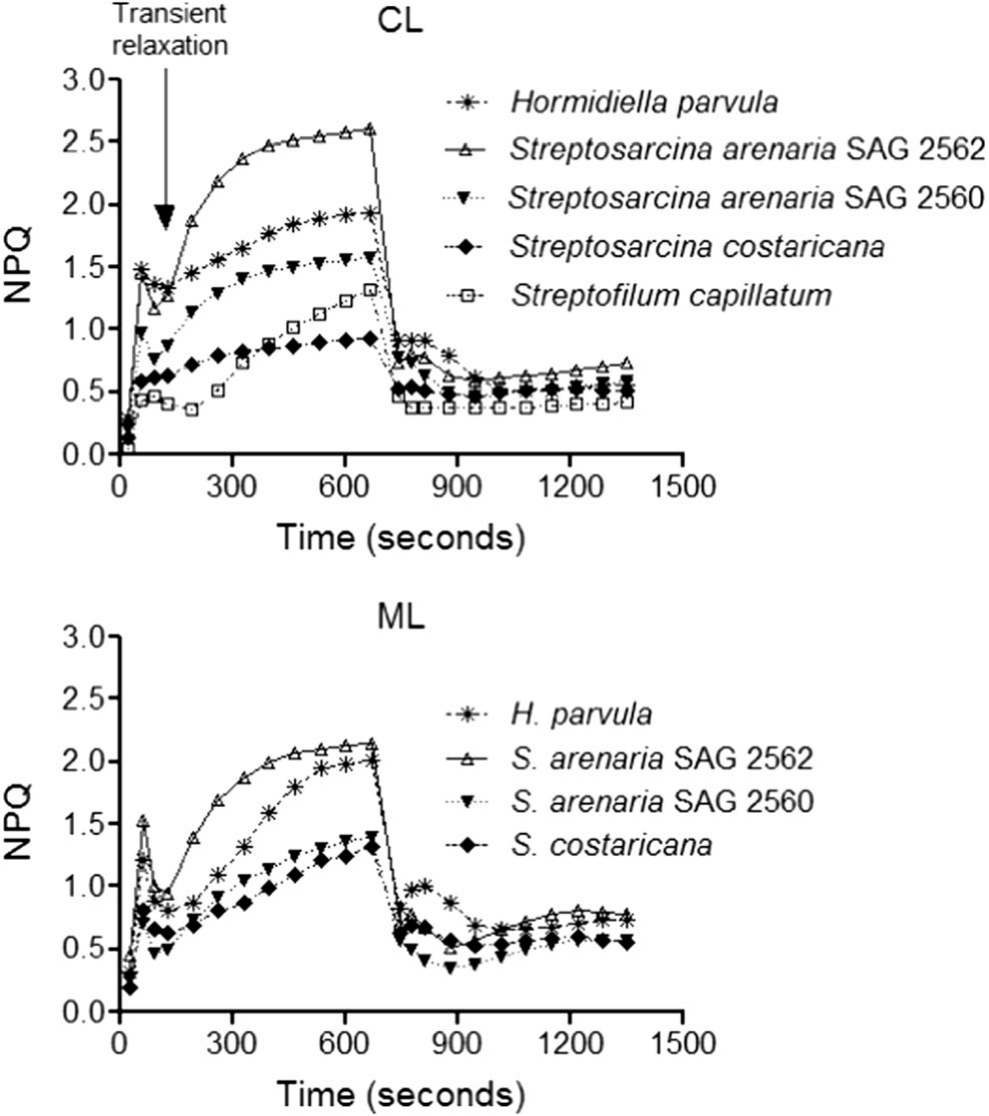
NPQ kinetics of *Hormidiella parvula* and *Streptosarcina arenaria* strains SAG 2562 and SAG 2560, *Streptosarcina costaricana* and *Streptofilum capillatum* cultured in control [CL; 60 μmol photons (PAR) m^−2^ s^−1^] and moderate [ML; 160 μmol photons (PAR) m^−2^ s^−1^] photon fluence rates. NPQ of *S. capillatum* under ML could not be determined, as the cultures died during the light-acclimation experiment. Arrow indicates the transient NPQ relaxation in the light. Data are from at least three culture replicates. Standard deviations are not shown

The maximal NPQ capacity (NPQ_max_) and NPQ kinetics of the four species grown in the two different light regimes are represented in Table 1 and Fig. 2. In CL, *S. arenaria* SAG 2562 showed higher maximal NPQ_max_ than *H. parvula*, *S. capillatum*, *S. arenaria* SAG 2562 and *S. costaricana* (one-way ANOVA, *p* < 0.0001). The NPQ_max_ of *H. parvula* was higher than *S. capillatum* and *S. costaricana*. The NPQ kinetics also showed a complex pattern. During the initial part of the activation phase, *H. parvula*, *S. capillatum* and both *S. arenaria* strains exhibited a transient relaxation of NPQ. Acclimation to ML enhanced NPQ_max_ in *S. costaricana* (*t* test, *p* = 0.0089) but no changes were measured in *H. parvula* (*t* test, *p* = 0.8008), *S. arenaria* SAG 2562 (*t* test, *p* = 0.0910) or *S. arenaria* SAG 2560 (*t* test, *p* = 0.4530).

### LHCSR3 Detection

An antibody against LHCSR3 failed to detect this protein in *S. arenaria* SAG 2562 and *S. costaricana* in western-blot analysis (Fig. S1). This antibody has been used for green algal species other than *Chlamydomonas* [30], but to our knowledge, no attempt has made to use it in streptophytes. Differences in the protein sequence between LHCSR3 in basal streptophytes such as *Klebsormidium nitens* and *Chlamydomonas* (only ~56% pairwise identity) clearly indicate the need to develop specific antibodies for these organisms.

### MAA Induction and Accumulation Under UVR

Members of the streptophyte genus *Klebsormidium* have been shown to produce MAAs [38]. In the species studied here, a UV-absorbing compound with a retention time of 4.3–4.4 min and an absorbance maximum at 324–325 nm was detected under both radiation conditions (PAR and PAR + UVA + UVB) (Table 2). This putative MAA exhibited identical chromatographic behaviour and features to that described in *Klebsormidium* [38], indicating the same chemical structure, which however is not yet elucidated. The steady-state concentrations of the putative MAA under PAR conditions ranged from 0.24 mg g^−1^ DW in *S. costaricana* to 6.91 mg g^−1^ DW in *H. parvula* (Table 2). After 4-day UV-A/B treatment, the MAA contents increased sharply in all isolates. Whilst strain *S. costaricana* synthesised up to 6.15 mg g^−1^ DW, *H. parvula* accumulated up to 16.06 mg g^−1^ DW (Table 2). The MAA induction factor after UVR exposure ranged from 2.2 to 25.6. In contrast to these four strains, *S. capillatum* exhibited a different putative MAA based on a much longer retention time (5.5 min) and an absorption spectrum with a slightly shorter maximum at 322–323 nm. Under control conditions, *S. capillatum* contained 0.16 mg g^−1^ DW of this unknown UV-absorbing compound, which rose to 1.24 mg g^−1^ DW, i.e. 7.6-fold, after UVR treatment (Table 2).

**Table 2:** The effect of 4-day treatment with 80–90 μmol photons m^−2^ s^−1^ PAR, 6–7 W m^−2^ UV-A and 0.37–0.45 W m^−2^ UV-B on the intracellular mycosporine-like amino acid contents (MAAs) in the streptophyte algal species studied

Species	PAR	PAR + UVA + UVB	Induction factor
*H. parvula*	6.91	16.06	2.3
S*. arenaria* SAG 2560	3.81	8.48	2.2
*S. costaricana*	0.24	6.15	25.6
*S. capillatum*	0.16	1.24	7.6

Due to a lack of biomass, only one replicate for each strain could be tested. Concentrations are given as mg MAAs g^−1^ dry weight

### Dehydration and Rehydration

**Fig. 3:**
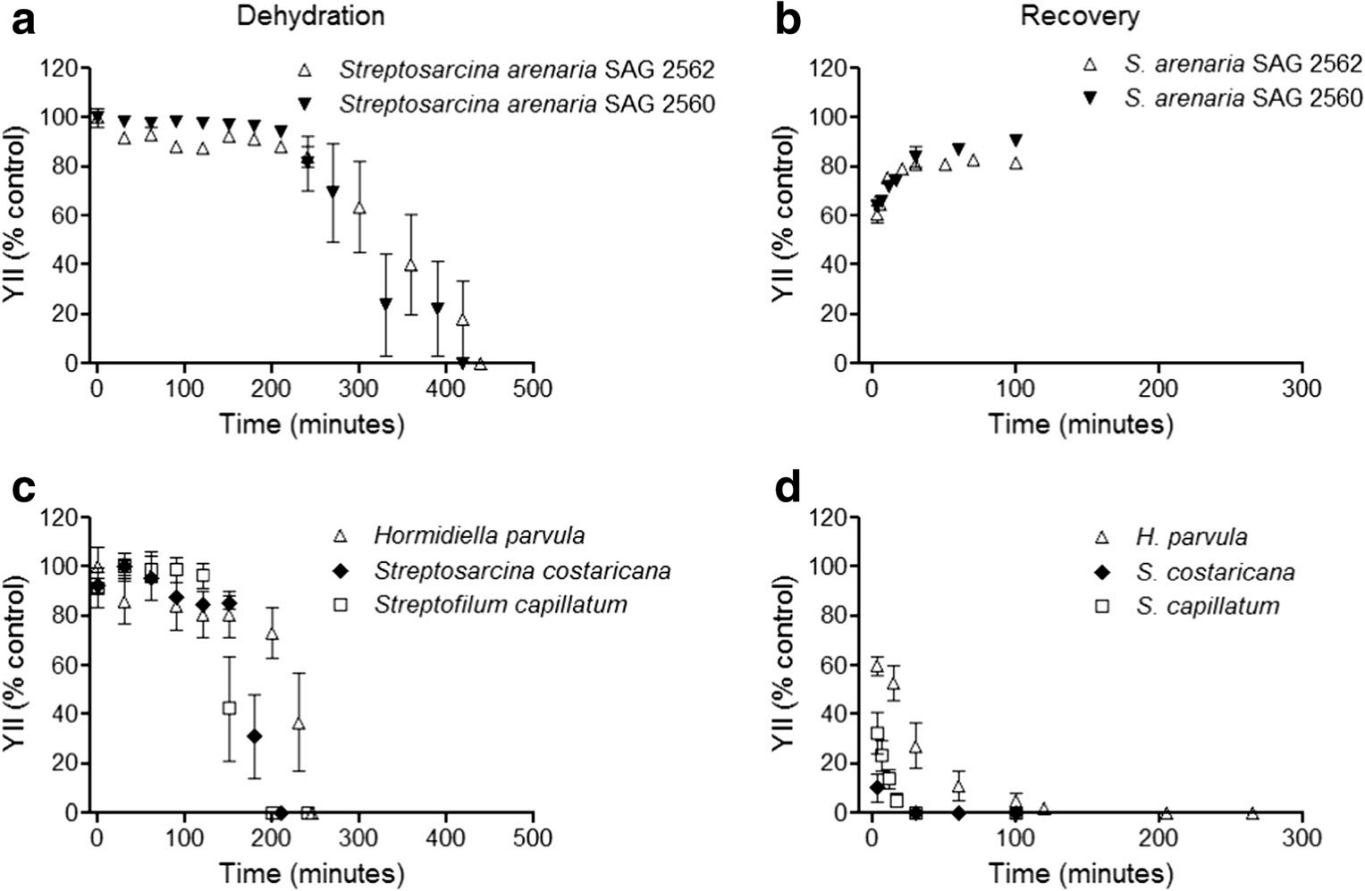
Changes in effective quantum yield (YII) during dehydration followed by rehydration with stock culture medium. **a**, **b** Results for dehydration and recovery of *Streptosarcina arenaria* strains SAG 2562 and SAG 2560. **c** Results for dehydration of *Hormidiella parvula*, *Streptosarcina costaricana* and *Streptofilum capillatum*. **d** No recovery of *H. parvula*, *S. costaricana* and *S. capillatum* after rehydration. Values are reported as mean of at least three independent measurements ± SD

The standardised methodological approach with the desiccation chambers and PAM measurements allowed regular comparative YII determinations in all streptophyte algal strains (Fig. 3). The data clearly indicated that the two closely related strains *S. arenaria* SAG 2562 and SAG 2560 exhibited a pronounced YII signal (ca. 90% of the control) for at least 210–240 min, before a threshold was reached, after which the YII slowly decreased to zero (after ca. 420 min) (Fig. 3a). Both strains were also capable of recovering YII within 30 min, up to > 80% of the control values (Fig. 3b). In contrast, *H. parvula*, *S. costaricana* and *S. capillatum* showed much earlier time points (120–150 min) until the YII thresholds were reached during dehydration, followed by a rather rapid YII decline to zero between 200 and 240 min (Fig. 3c). After rewetting, these species did not recover, at least within 100 min (Fig. 3d). *H. parvula* was even followed for 1355 min, but still no signal for YII could be recorded. However, taking into consideration that *H. parvula*, *S. costaricana* and *S. capillatum* are also from terrestrial habitats, a tolerance to milder dehydration than that imposed in this study could be expected for these species.

### Photosynthesis and Respiration Response to Short-Term Rise of Temperature

**Fig. 4:**
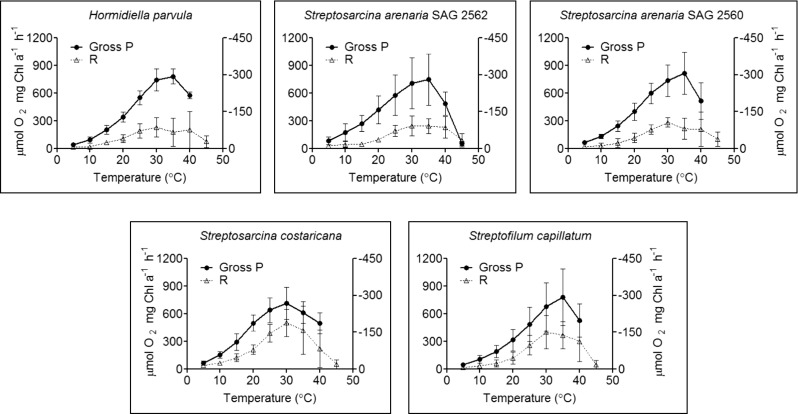
Rapid thermal responses of gross photosynthesis (P) and respiration (R) for the species studied. At 45 °C, gross P was completely suppressed for *Hormidiella parvula*, *Streptosarcina arenaria* SAG 2560, *Streptosarcina costaricana* and *Streptofilum capillatum*. Measurements were performed on at least three independent culture replicates

**Fig. 5:**
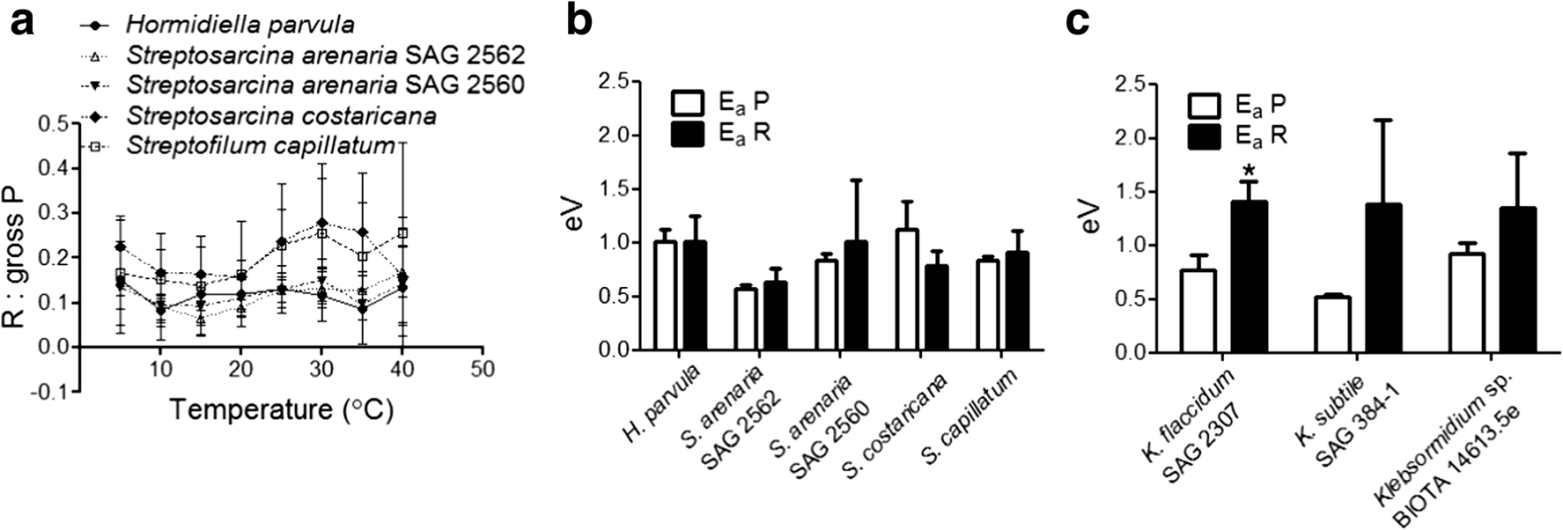
**a** Respiration (R) to gross photosynthesis (P) ratios as function of increasing temperature. **b** Comparison between activation energies for gross photosynthesis (E_a_ P) and respiration (E_a_ R) for *Hormidiella*, *Streptosarcina* and *Streptofilum*. **c** Comparison of activation energies measured for *Klebsormidium* spp. and calculated from results reported by Karsten et al. [16, 60]. Asterisks indicate statistically significant differences of E_a_ R in comparison to E_a_ P. Values are calculated from at least three independent measurements

The regulation of gross photosynthetic capacity and respiration, for these species of basal streptophytes exposed to a rapid increase of temperature, are shown in Fig. 4. In relation to gross photosynthesis, no species-specific response was observed (two-way ANOVA, *p* = 0.7391). For all species, increasing the temperature from 5 °C to optimal (*T*_*h*_, *T*_opt_; Table 3) positively regulated rates of gross photosynthesis (two-way ANOVA, *p* < 0.0001). In relation to respiration, the increase with temperature (two-way ANOVA, *p* < 0.0001) was species-specific (two-way ANOVA, *p* = 0.0011): *S. costaricana* showed a higher respiration rate than *S. arenaria* SAG 2562 and *H. parvula* at 30–35 °C. Above optimal temperatures, both metabolic processes rapidly declined. At 45 °C, gross photosynthesis was completely suppressed for *H. parvula*, *S. arenaria* SAG 2560, *S. capillatum* and *S. costaricana*. In spite of the regulation of the photosynthetic and respiration rates, the respiration to gross photosynthesis ratio (R:gross P) remained unchanged as the cells warmed (Fig. 5a; two-way ANOVA, *p* = 0.1114). These R:gross P ratios described respiration as between 7 and 28% of the gross P, which is in line with average values (10–15%) previously reported for phytoplankton species [59]. The parameters describing the temperature dependence of gross photosynthesis and respiration are shown in Fig. 5b and Table 3. The activation energy for photosynthesis (E_a_ P) was lower for *S. arenaria* SAG 2562 than for *H. parvula* and *S. costaricana* (one-way ANOVA, *p* = 0.0055). The T_h_ for photosynthesis (T_h_ P) was lower for *S. costaricana* in comparison to *S. capillatum* and *S. arenaria* SAG 2562 (one-way ANOVA, *p* = 0.0012). However, the differences of E_a_ P and T_h_ P between *S. arenaria* SAG 2562 and the other species may be related to mathematical reasons, since the value of gross photosynthesis at 45 °C was included in the fitting through the Sharpe-Schoolfield equation, rather than to a biological significance. Since gross photosynthesis was not measurable at 45 °C for *H. parvula*, *S. arenaria* SAG 2560, *S. costaricana* or *S. capillatum*, the de-activation energy and optimal temperature for photosynthesis (E_h_ P, T_opt_ P) parameters were not calculated. In relation to respiration, no differences amongst species were found for E_a_ R (one-way ANOVA, *p* = 0.3758), E_h_ R (one-way ANOVA, *p* = 0.2984), T_h_ R (one-way ANOVA, *p* = 0.3627) or T_opt_ R (one-way ANOVA, *p* = 0.3494). In all species, E_a_ R was not statistically different from E_a_ P. In *S. costaricana*, the T_h_ P was lower than the T_h_ R (*t* test, *p* = 0.0295). As a relative comparison with other basal streptophytes, we added parameters describing the temperature dependence of gross photosynthesis and respiration for *Klebsormidium* spp. obtained from the results of Karsten et al. [16, 60] (Fig. 5c and Table 3). For *K. flaccidum* (Kützing) Silva, Mattox et Blackwell (SAG 2307), E_a_ P was ~1.8-fold lower than E_a_ R (*t* test, *p* = 0.0089). Although not statistically significant (*t* test, *p* = 0.1280), the E_a_ P was also ~2.6-fold lower than E_a_ R in *K. subtile* (Kützing) Mikhailyuk, Glaser, Holzinger & Karsten (SAG 384–1). For *Klebsormidium* sp. (BIOTA 14613.5e), no difference in E_a_ P and E_a_ R was found (*t* test, *p* = 0.2384).

**Table 3:** Parameters extrapolated from the thermal responses of gross photosynthesis and respiration (Fig. 4), using natural log-transformed metabolic rates fitted through the modified Sharpe-Schoolfield equation for high-temperature inactivation [51, 53]

	Gross photosynthesis				Respiration			
Species	*T*_opt_ (°C)	*T*_*h*_ (°C)	*E*_*a*_ (eV)	*E*_*h*_ (eV)	*T*_opt_ (°C)	*T*_*h*_ (°C)	*E*_*a*_ (eV)	*E*_*h*_ (eV)
*H. parvula*		30 (0)	1.01 (0.10)		31 (5)	31 (7)	1.01 (0.24)	2.46 (0.89)
*S. arenaria* SAG 2562	36 (1)	39 (1)	0.57 (0.03)	5.07 (0.65)	36 (2)	38 (3)	0.63 (0.13)	3.89 (1.69)
*S. arenaria* SAG 2560		32 (2)	0.83 (0.07)		32 (3)	34 (5)	1.01 (0.57)	2.36 (0.37)
*S. costaricana*		24 (6)*	1.12 (0.26)		33 (4)	35 (5)	0.78 (0.14)	4.44 (1.92)
*S. capillatum*		34 (1)	0.83 (0.04)		35 (3)	37 (4)	0.91 (0.20)	4.85 (2.58)
*K. flaccidum*	34 (1)	37 (2)	0.76 (0.14)*	3.89 (1.01)*	37 (2)	29 (7)	1.41 (0.19)	1.82 (0.41)
*K. subtile* ^a^	35 (1)*	38 (0)	0.52 (0.04)	6.95 (3.11)	38 (1)	32 (11)	1.38 (0.78)	2.10 (0.15)
*Klebsormidium* sp.^a^	34 (1)*	36 (2)	0.92 (0.10)	6.60 (2.53)*	37 (1)	34 (7)	1.35 (0.52)	2.34 (0.21)

Asterisks indicate statistically significant differences of the parameters for gross photosynthesis relative to respiration. Values in brackets represent standard deviation (*n* ≥ 3)

*T*_*opt*_ optimal temperature, *T*_*h*_ temperature at which half of the enzymes are inactive, *E*_*a*_ activation energy, *E*_*h*_ de-activation energy

^a^Parameters were analysed from temperature curves reported in Karsten et al. [16, 60]

## Discussion

This study investigated how the photosynthetic apparatus of recently described members of basal streptophyte algae responds to changes of radiation (both PAR and UVR), water availability and temperature. The following results were obtained: (1) differences in photosynthetic traits, photoprotective capacities and abilities to photoacclimate to higher light regimes amongst the investigated taxa; (2) species with constitutive low-light requirements for photosynthesis exhibited a greater dehydration tolerance, providing further evidence that desiccation is a physiological driver that shapes adaptation to low light; and (3) in *Hormidiella*, *Streptosarcina* and *Streptofilum*, rates of photosynthesis and respiration exhibited similar temperature requirements, contrasting with related streptophytes such as members of the genus *Klebsormidium*, and highlighting diverse degrees of thermal sensitivity amongst terrestrial microalgae.

As in other basal terrestrial streptophytes [16, 22, 48], the photosynthetic apparatus of all species studied here required only low light levels for photosynthesis. This widespread ability of basal terrestrial streptophytes to function effectively under low light may confer an advantage on these organisms in micro-environments of biological soil crusts, where incident light availability declines sharply, even on a micro-scale, with depth [46, 61]. However, our results showed that whereas the low-light requirement was constitutive for *S. arenaria*, other species, i.e. *H. parvula* and *S. costaricana*, were able to reorganise the photosynthetic apparatus and optimise photosynthesis in a higher-light environment. At a functional level, the limited degree of acclimation in both *S. arenaria* strains can be interpreted as the preference of this species to maintain homoeostasis of the photosynthetic apparatus, which could help to avoid the capital costs related to acclimation [62–64]. In contrast, the higher rETR_max_ and lower α or β in *H. parvula* and *S. costaricana* grown in ML reflect photosynthetic adjustments such as lowering pigment contents and decreases in PSII, PSI numbers and/or their antenna size [62, 65–68].

In relation to NPQ, different responses have been observed during exposure of green algae to higher-light regimes [69]. In the case of basal streptophytes, whereas exposure to high light may enhance NPQ capacity for *Klebsormidium* [23] and *S. costaricana*, for others such as *H. parvula* and *S. arenaria* strains, the NPQ capacity remained unaltered. This unchanged NPQ capability during light acclimation indicates that the NPQ is involved in protecting the photosynthetic apparatus only during short-term light changes [68]. This might also explain why species such as *S. arenaria* possess constitutive low-light requirements for photosynthesis. Nevertheless, we cannot exclude that a longer acclimation time or higher photon fluence rates might be necessary to cause quantitative NPQ modification [68, 70]. Mechanistically, the rapid NPQ relaxation in the dark indicates that the energy-dependent component qE is the major portion of the NPQ [71], and its activation, as in other basal streptophytes, is likely to be regulated by LHCSR proteins and lumen acidification [23]. Although an antibody against LHCSR3 failed to recognise this protein in *S. costaricana* and *S. arenaria* SAG 2562, we do not exclude the possible presence of different LHCSR isoforms in these basal streptophytes [23, 72]. Further analysis is necessary to clarify the involvement of PSBS in LHCSR function also in streptophyte algae, as suggested in Chlamydomonadales (Chlorophytes) [32, 73]. Besides qE, the NPQ kinetics suggests that additional mechanisms are involved in NPQ activation. As in the case of *C. reinhardtii* (in high-light conditions), dark incubation before NPQ measurements may activate chlororespiration and reduce the PQ pool (inducing a state II transition) [57, 74]. The transient NPQ relaxation, observed in the initial phase of the NPQ induction, could indicate the occurrence of rapid oxidation of the PQ pool and relocation of the light-harvesting complex II from PSI to PSII, i.e. state II to I transition [57].

In addition to NPQ, the presence of UVR protecting compounds such as MAAs may further modulate species tolerance under natural insolation [38]. MAAs function as passive shielding solutes by dissipating the absorbed UVR energy in the form of harmless heat, without generating photochemical reactions [41]. These compounds typically show high molar absorptivity for UV-A and UV-B, and they are photochemically stable molecules, both of which are prerequisites for their sunscreen function [75, 76]. *H. parvula*, *S. arenaria* strain SAG 2560 and *S. costaricana* synthesised and accumulated a specific putative MAA, which matched those recently described in various isolates of the closely related *Klebsormidium* and *Interfilum* [77]. In contrast, *S. capillatum*, the streptophyte from a separate basal lineage, contained another rather unusual putative MAA which did not match with any other related taxa in terms of retention time and absorbance maximum. It is reasonable to assume that this UV-absorbing compound found in *S. capillatum* could be a new, so far chemically unidentified MAA. The capability of accumulating specific sunscreen compounds, regardless of their biochemical nature, indicated that all species studied have protective mechanisms against potential UVR damage.

Supporting our hypothesis, species-specific photoacclimation abilities were also linked to dehydration tolerance. *H. parvula* and *S. costaricana*, which acclimated to higher light regimes, were not capable of tolerating desiccation. In contrast, both *S. arenaria* strains, with constrained low-light requirements for photosynthesis, were able to tolerate dehydration. Previous investigations on *Klebsormidium* sp. and other desert green algae showed that exposure to relatively high photon fluence rates during dehydration compromises the function of the photosynthetic apparatus as well as the ability to recover when water is again available [22, 46]. This points to the physiological problem that if terrestrial algae can acclimate and occur under high-light conditions, they might be at risk if water availability in the environment is fluctuating and falls below the cellular optimum. Contrariwise, lower-light adaptation and occurrence in shaded conditions may confer an advantage in habitats with frequent dehydration-rehydration cycles. Overall, these results support a previous statement that dehydration is a selective force shaping the adaptation of members of Klebsormidiophyceae to low light [22].

One explanation for the conspicuously different dehydration tolerances amongst *S. arenaria* and *H. parvula*, *S. costaricana* and *S. capillatum* could be related to their cell morphology. Both *S. arenaria* strains SAG 2562 and SAG 2560 are characterised by cell packets, whilst the other species occur as filaments or filaments that easily disintegrate into single cells [9]. These data are in agreement with those on various species of *Interfilum* [48], which indicate that single terrestrial algal cells that are strongly associated with other algal cells in an aggregate, colony or even biofilm are well protected against water loss. The formation of such cell packets could be related to self-protection of cells in aggregate, as well as to a joint matrix of extracellular polysaccharides (EPS; mucilage) in which the cells are embedded, thus preventing dehydration, at least partly, and hence maintaining physiological processes [78]. The freshwater *Coleochete* species showed under simulated terrestrial culture conditions a strong change in morphology from a typical radial thallus to the formation of packet-like structures [79]. Therefore, the formation of this morphotype might be a beneficial mechanism for algae to thrive under terrestrial conditions.

Although the five basal streptophytes showed different light-acclimation abilities and dehydration tolerances, they showed preferences for similar temperature conditions. Their short-term thermal responses of photosynthesis and respiration are comparable to other algal or plant species, exponentially increasing up to an optimum and followed by a rapid decline [51, 52, 80, 81]. This reflects the temperature sensitivity of electron-transport components and enzymatic machinery involved in both metabolic processes [80, 82–85]. Interestingly, the analogous maximal light-saturated rates of photosynthesis (*P*_max_), which is limited by Calvin-Benson cycle activity [86], indicate that *Hormidiella*, *Streptosarcina* and *Streptofilum* have similar Rubisco carbon-fixation capacities at various temperatures.

Comparing photosynthesis to respiration, the unchanged R:gross P during the temperature-response curves, together with an E_a_ P similar to E_a_ R, showed that photosynthesis and respiration have the same temperature requirements in *Hormidiella*, *Streptosarcina* and *Streptofilum*. In comparison, for members of sister lineages such as *Klebsormidium*, E_a_ P is lower than E_a_ R, indicating that photosynthesis is less sensitive to temperature than is respiration. The reasons, advantages and/or disadvantages for having a similar or a different thermal sensitivity between photosynthesis and respiration amongst organisms that are phylogenetically related or occupy apparently similar terrestrial niches are not yet clear. Physiologically, the ability to maintain a balanced photosynthesis and respiration could allow algae to maintain efficient carbon allocation to cell growth [87, 88] when rapid (e.g. diurnal) temperature changes occur. For *Klebsormidium* spp., the higher temperature dependence of respiration in comparison to photosynthesis indicates a higher carbon gain at lower temperatures [13, 21]. In the case of *K. subtile* (SAG 384–1), this trait may reflect a cold adaptation of this species, which is in line with its arctic habitat [16, 51]. In ecological terms, the occurrence of algal taxa with similar metabolic temperature requirements could contribute to maintain the metabolic balance of ecosystems (e.g. within cryptogamic covers) over a range of temperature conditions [89, 90]. An equal or different temperature dependence of photosynthesis and respiration may be particularly important for physiological responses to global warming, where species-specific adaptations of both metabolic processes are likely to occur [54, 88].

## Conclusions

The present study demonstrated that basal streptophytes inhabiting aero-terrestrial environments possess different photosynthetic traits and photoprotective capacities. Moreover, low-light requirements for photosynthesis are not always constitutive, and for some of the species investigated here, higher photosynthetic rates can be reached with acclimation to higher photon fluence rates. Constitutive low-light requirements were coupled with dehydration tolerance, supporting the concept expressed earlier [22] that low-light adaptation traits in Klebsormidiophyceae are shaped by dehydration. The rather similar temperature requirements for photosynthesis and respiration conspicuously distinguished the *Hormidiella*, *Streptosarcina* and *Streptofilum* strains investigated from other terrestrial streptophytes, emphasising an important interspecific variability in metabolic thermal response. Overall, this physiological diversity characterises streptophyte species that are suited for different spatial and temporal terrestrial conditions. For instance, taxa that are more restricted to low light could exploit deeper parts of biological soil crusts, where less light is available [61], or occur in later successional stages of soil colonisation, e.g. in a rather mature community, when light shading is provided by other species. Besides light, seasonal or future changes of both temperatures and water regimes [91, 92] could select and control the distribution of species with similar or different temperature-requirements for photosynthesis and respiration, or tolerance to dehydration.

## Electronic Supplementary Material

ESM 1 (PPTX 110 kb)
