# Supplementary material for: Light and Dehydration but Not Temperature Drive Photosynthetic Adaptations of Basal Streptophytes (Hormidiella, Streptosarcina and Streptofilum) Living in Terrestrial Habitats
Source: Microb Ecol. 2018 Jul 4;77(2):380–93. doi: 10.1007/s00248-018-1225-x (PMC6394494; doi:10.1007/s00248-018-1225-x)
Supplement: Supplementary file 1 — (PPTX 110 kb) [file 248_2018_1225_MOESM1_ESM.pptx]

## Slide 1
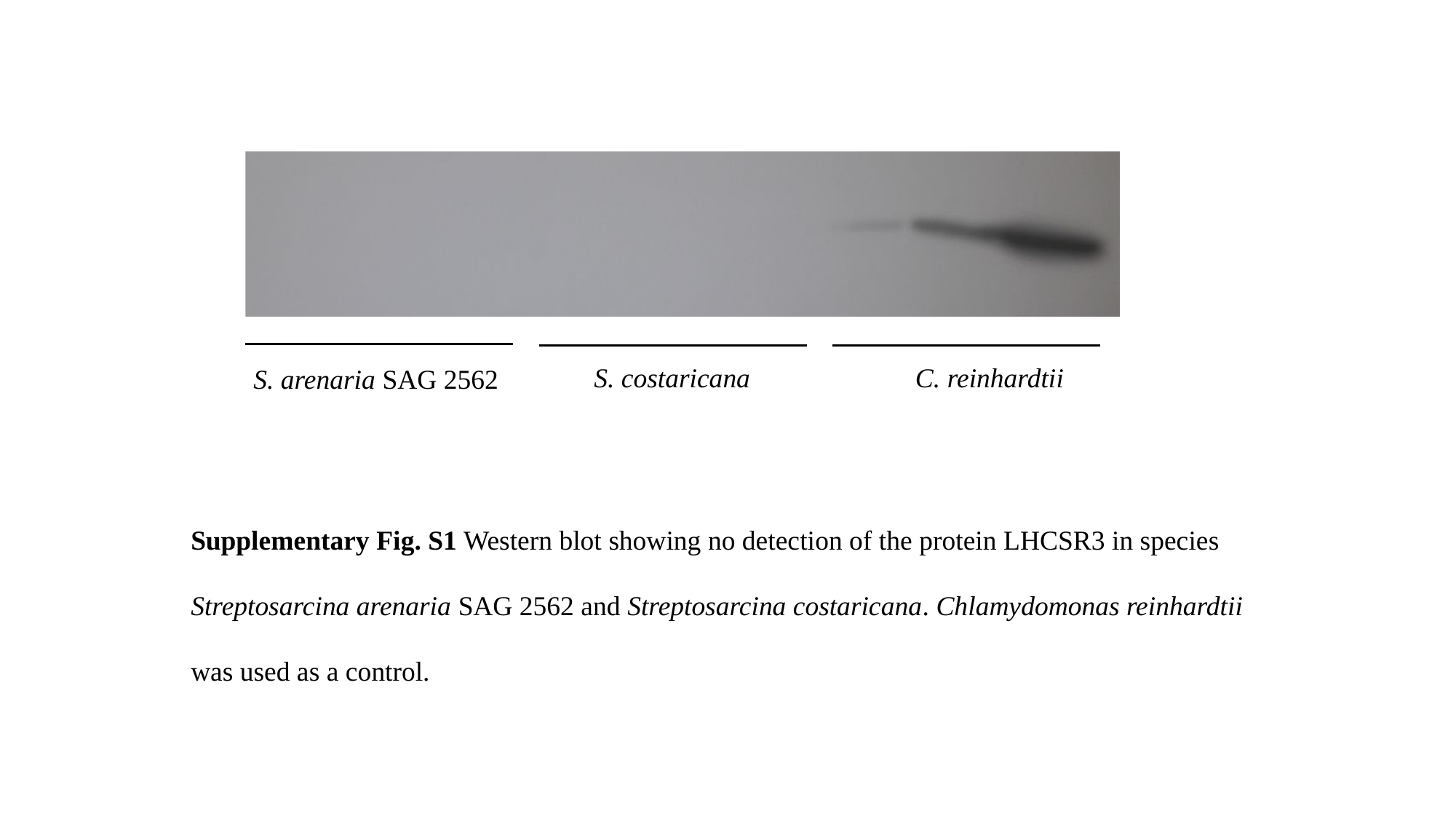

S. costaricana
C. reinhardtii
S. arenaria SAG 2562
Supplementary Fig. S1 Western blot showing no detection of the protein LHCSR3 in species
Streptosarcina arenaria SAG 2562 and Streptosarcina costaricana. Chlamydomonas reinhardtii
was used as a control.
